# Multiple lung metastases presenting as ground-glass opacities in a pulmonary adenocarcinoma: a case report

**DOI:** 10.1186/1757-1626-2-6910

**Published:** 2009-05-29

**Authors:** Noriko Yanagitani, Kyoichi Kaira, Tamotsu Ishizuka, Haruka Aoki, Mitsuyoshi Utsugi, Yasuo Shimizu, Noriaki Sunaga, Katsuaki Endou, Takeshi Hisada, Masatomo Mori

**Affiliations:** Department of Medicine and Molecular Science, Gunma University Graduate School of Medicine3-39-15, Showa-machi, Maebashi, Gunma 371-8511Japan

## Abstract

**Introduction:**

Focal ground-glass opacity on computed tomography suggests several disorders including inflammatory disease, fibrosis, or a primary lung neoplastic lesion, metastatic lung tumor.

**Case presentation:**

The case of a 55-year-old female presenting with adenocarcinoma of the lung is herein reported. Computed tomography of the chest revealed a primary mass lesion in the upper lobe of the right lung and multiple metastases presenting as ground-glass opacities. Macroscopic metastases were observed in the bone, the hilar and mediastinal lymph nodes, and another lobe. This case was advanced lung cancer. We assumed that the multiple ground-glass opacity lesions were metastasis in the lungs. Chest CT revealed a partial response of the primary site and the multiple ground-glass opacities after systemic chemotherapy.

**Conclusion:**

A metastatic lung tumor showing ground-glass opacity is uncommon. It is quite difficult to distinguish between multiple primary lung cancers and intrapulmonary metastasis when patients present with multiple lung nodules. A lot of clinical information is therefore required to make an accurate diagnosis in such cases.

## Introduction

Some lung cancers are characterized by the unique sign of ground-glass opacity (GGO) on high resolution computed tomography (HRCT) scanning. Although, it is quite difficult to preoperatively distinguish between multiple lung cancers and intrapulmonary metastasis. We herein report a case of lung cancer which revealed various image views. The primary site showed a very large tumor, whereas metastasis of the bilateral lungs showed GGOs. Chest CT revealed a partial response of the primary site and the multiple GGOs after systemic chemotherapy. Therefore, we concluded that the multiple GGOs represented intrapulmonary metastasis in a patient with advanced lung cancer.

## Case presentation

**Figure 1.**
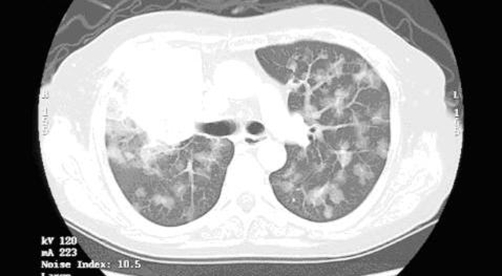
Chest CT showing a mass of the right upper lobe and multiple pure ground-glass opacity of the bilateral lungs.

**Figure 2.**
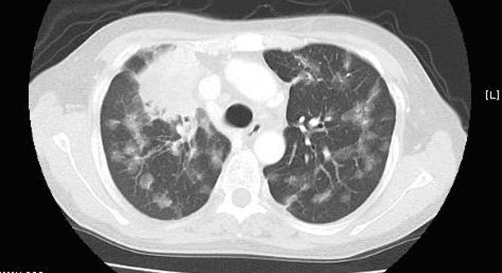
Chest CT showing a partial response of the primary site and multiple metastases of the bilateral lungs.

A 55-year-old, Japanese, female housewife, was admitted to the hospital with a 1-month history of cough and weight loss. She had two children and no significant medical history or family history. Her physical examination was unremarkable. Computed tomography (CT) of the chest revealed a 4 cm mass lesion in the upper lobe of the right lung, mediastinal lymph nodes, and multiple ground-glass opacities (GGO) in the bilateral lungs (Figure 1). A transbronchial lung biopsy (TBLB) revealed an adenocarcinoma. Her serum carcinoembryonic antigen (CEA) and neuron-specific enolase (NSE) levels were 60.6 ng/ml (normal range <2.5 ng/ml) and 16.4 ng/ml (normal range <12.0 ng/ml), respectively. Bone scintigraphy revealed an increased uptake in multiple bones. The patient was diagnosed to have c-stage T4N2M1 disease. She was treated with platinum-based chemotherapy in December 2005. After four cycles of chemotherapy, chest CT revealed a partial response of the primary site, lymph nodes metastasis, and multiple GGO. On May 2006, chest CT revealed evidence of the marked growth of the primary site, lymph nodes and multiple GGO. A recurrence of lung cancer was confirmed. Since the patient had deletion mutations in exon 19 of EGFR (del E747-S753), she was treated with gefitinib (Iressa) (250 mg/day) every day. This led to a rapid improvement in the clinical symptoms and a chest CT scan revealed a partial response of the primary site, lymph nodes and multiple GGO (Figure 2). In October 2006, however, there was a marked growth of the primary site, lymph nodes and multiple GGOs. Although she was treated with amrubicin, the treatment was not effective. No follow-up therapy was performed. Her condition deteriorated and she died due to the progression of lung cancer two months after presentation.

## Discussion

CT imaging provides us with increasing opportunities to find multiple small pulmonary nodules, which sometimes appear as GGO. It is often difficult to distinguish between multiple primary lung cancers and intrapulmonary metastasis by radiology, when patients present with multiple lung nodules. However the diagnosis is quite critical for deciding on the clinical strategy for lung cancers. Tsushima *et al.* [1] reported that radiographic findings in one patient strongly suggested that multiple lung nodules were multiple primary adenocarcinomas, which was confirmed by histopathology. This patient showed primary lung cancer with multiple GGO on thoracic CT scan. In the present case, it was difficult to determine whether the multiple GGO demonstrated metastasis of the primary lung cancer or multiple lung cancers, because there were multiple lung nodules in the bilateral lungs and it was impossible to completely discriminate the multiple lung nodules. Although some cases of metastatic lung tumor showing GGOs from adenocarcinoma of the gastrointestinal tract and malignant melanoma have been reported, GGO due to a metastatic tumor is uncommon [2]. Generally, the differential diagnosis of focal GGO should include inflammatory diseases, focal fibrosis, atypical adenomatous hyperplasia, and adenocarcinoma. However, it is difficult to make a diagnosis of multiple GGO by radiographic examinations. In the current case, chest CT revealed a partial response of the primary site and the multiple GGOs after systemic chemotherapy. Therefore, we concluded that the multiple GGOs were intrapulmonary metastasis in the advanced lung cancer. The possibility of metastatic lung tumors should thus be suspected in a patient with a history of malignancy who demonstrates a rapid growth of multiple GGO lesions.

## Conclusions

This report describes a case of a metastatic lung tumor showing GGO on CT in an advanced non-small cell lung cancer patient, although the occurrence of metastatic lung tumors showing GGO is uncommon.

## Abbreviations

CEA: carcinoembryonic antigen
CT: computed tomography
GGO: ground-glass opacity
HRCT: high resolution CT
NSE: neuron-specific enolase
TBLB: transbronchial lung biopsy
